# Veteran Reports of Anxiety and Depression Before, During, and After COVID-19: Associations With Race/Ethnicity, Gender, and Traumatic Exposures

**DOI:** 10.1155/da/5572394

**Published:** 2025-09-05

**Authors:** Ryan Chesnut, Keith R. Aronson, Daniel F. Perkins

**Affiliations:** ^1^Clearinghouse for Military Family Readiness, Pennsylvania State University, University Park, Pennsylvania, USA; ^2^Social Science Research Institute, Pennsylvania State University, University Park, Pennsylvania, USA; ^3^Department of Biobehavioral Health, Pennsylvania State University, University Park, Pennsylvania, USA; ^4^Department of Agricultural Economics, Sociology and Education, Pennsylvania State University, University Park, Pennsylvania, USA

## Abstract

The COVID-19 pandemic was a world-wide health emergency that resulted in individuals experiencing challenges in numerous life domains. Life domains affected included physical and mental health, finances, and social isolation. Many health and research professionals evidenced concern that veterans were more likely than civilians to experience COVID-19 related problems due to their “at-risk” health status. Veterans are at-risk for health problems due to encountering unique military experiences such as traumatic exposures, development of trauma-related mental health symptoms or disorders, combat-related injuries, and disability, exposure to toxins such as burn pits and biological agents, and living with chronic stress during their transition to civilian life. It was suggested that the disruptions and challenges the COVID-19 pandemic created could trigger mental health problems among veterans. Indeed, based on cumulative stress theory, female veterans and veterans from racial and ethnic minority groups were thought to be particularly vulnerable for experiencing mental health challenges. The current study examined changes in the symptoms of depression and anxiety before, during, and after the COVID-19 pandemic among a large and diverse sample of post-9/11 veterans. As predicted, when compared to White male veterans, male and female veterans from racial and ethnic minority groups reported having higher symptom levels of anxiety and depression before, during, and after the COVID-19 pandemic. All veterans, except for females from racial and ethnic minority groups, reported experiencing increases in symptoms over time. Exposure to adverse childhood experiences (ACEs), combat exposure, and length of longest deployment were inconsistently associated with symptoms over time. The results suggest that the COVID-19 pandemic was associated with individuals experiencing increased anxious and depressive symptoms over time, although not in a wholly consistent manner. Future global health emergencies may have differential gender- and race/ethnicity-based effects on veterans; thus, veteran-serving organizations should carefully plan their responses to such crises.

## 1. Introduction

The COVID-19 pandemic (heretofore referred to as COVID-19) revealed in stark terms the devastation that can be wrought by a global pandemic. The pandemic was associated with high stress exposure, psychological problems, severe illness, chronic declines in functioning, and in some cases, death [1, 2]. In addition, the pandemic spread fear across the United States, and many individuals experienced the pandemic as traumatic [3]. A recent meta-analysis of 59 studies found that men and individuals aged 70 years and older were at significantly elevated risk for infection, severe COVID-19 disease, higher admittance to intensive care, and higher mortality rates than women and younger people, respectively [4]. Other studies demonstrated that racial/ethnic minorities bore a disproportionate disease burden [5]. Furthermore, United States military veterans were found to carry excess infection rates, morbidity, and mortality during the pandemic compared to non-veterans [6, 7], particularly among Veterans from racial and ethnic minority groups [8]. It is not clear if female veterans bore a disproportionate burden of COVID-19, although there is some evidence that female veterans from racial and ethnic minority groups were at elevated risk for infection relative to their White female peers [9, 10]. These studies of Veterans and COVID-19 used samples comprised of Veterans of all wars and not just those Veterans classified as serving during the Global War on Terror, which began in 2001 (i.e., post-9/11 veterans).

Veterans are considered an at-risk population due to pre-, during-, and post-military service factors that distinguish them from civilians [11]. For example, veterans are approximately 25% more likely than civilians to report exposure to adverse childhood experiences (ACEs) [12], such as physical, emotional, and sexual abuse [13]. Exposure to ACEs is a significant risk factor for the development of adult mental health problems and premature mortality [13–16]. During military service, many veterans are exposed to combat (and other traumatic experiences), and these exposures can increase a veteran's likelihood of developing anxiety, depression, substance use, posttraumatic stress disorder (PTSD), moral injury, serious physical injury, and disability [15, 17–19]. After military service, veterans must reintegrate to civilian life, which could include reengaging with families and communities, finding work, and pursuing further education [20]. Veterans must also contend with losing their military identity, regimentation, and close military friends and colleagues [21]. Between 16% and 42% of veterans report they struggle with reintegration [22, 23]. Given that many of these problems are chronic and ongoing, veterans carry lifelong risks [11, 19].

### 1.1. Cumulative Stress Theory, COVID-19, and Post-9/11 Veteran Mental Health

Research has repeatedly found that, when psychosocial stressors accumulate over time, these stressors are likely to have substantial impacts on an individual's physical and mental health [24]. Exposure to a multitude of stressors or repeated exposure to the same stressor can create deleterious health impacts [24]. Cumulative stress theory discusses the effects of recurring and accruing exposure and is particularly relevant to those in the military. Service members and veterans experience the typical stressors that civilians do and unique stressors such as frequent separations, multiple relocations, and combat exposure [25]. Females [26] and people from racial/ethnic minorities [27] in the military report experiencing significantly higher stressor exposures than their White male peers. Moreover, during military-to-civilian transitions (MCTs), veterans can be simultaneously faced with a variety of stressors, such as finding a place to live, making social connections, obtaining employment, loss of military identity, and fitting into new communities [28].

Cumulative stress research has also been used to understand and explain the poorer health experienced by ethnic/racial minorities [29]. People from ethnic/racial minority groups often face the chronic stress of racism, discrimination, and exclusion [30], and experience persistent social disparities of health [31]. During the pandemic, racial/ethnic minority individuals were more likely to contract COVID-19 and encounter more barriers to healthcare [32]. Indeed, a recent study found that female veterans and veterans from racial/ethnic minority groups reported more stress than their White male peers in the areas of mental health, employment, and financial problems during COVID-19 [1].

During COVID-19, women reported higher levels of stress than men. Females were more likely to be considered “essential” employes in the healthcare field during the pandemic [33], and they provided a disproportionately higher level of child care and faced a higher level of work–family demands [34]. Women also experienced higher levels of unemployment and financial problems compared to men during COVID-19 [35]. In addition, female veterans were at risk for psychological stress during the pandemic because they have higher rates of experiencing prior traumas [36], including ACEs [14].

#### 1.1.1. The Present Study

Utilizing cumulative stress theory, this study examined changes in the psychological symptoms of a large sample of post-9/11 veterans from before to after COVID-19. For this study, the authors hypothesized that (a) all post-9/11 veterans would report experiencing increasing mental health symptoms from before to after COVID-19 and (b) female veterans and veterans from racial and ethnic minority groups, compared to their White male peers, would evidence higher levels of mental health symptoms before, during, and after COVID-19. In addition, greater ACEs, combat exposure, number of deployments, and maximum deployment length are predicted to be associated with increased mental health symptoms at each point in time.

## 2. Methods

### 2.1. Participants and Procedures

Study data came from Waves 1, 5, and 6 of The Veterans Metrics Initiative (TVMI) and Waves 7 and 8 of the Veterans Engaging in Transitions Study (VETS), an extension of TVMI. TVMI was a large-scale, longitudinal, survey-based study of post-9/11 veterans' transitions from military to civilian life. Study design details and sample demographics have been previously published [37, 38]. TVMI used the Department of Veterans Affairs/Department of Defense Identity Repository (VADIR) to determine the population of all veterans who had separated from the military within a 3-month window from August to October 2016 (*N* = 48,965). Study invitations were sent to all identified veterans, and 19.5% (*n* = 9566) completed the Wave 1 baseline survey administered in November 2016 [37]. Surveys were re-administered at 6-month intervals to all veterans who completed Wave 1 for a total of six waves that spanned 2.5 years. The Wave 5 survey was administered in November 2018 and had a 61.1% retention rate (*n* = 5844). The Wave 6 survey was administered in May 2019, approximately 10 months before the World Health Organization (2019) declared COVID-19 a pandemic and had a 55.0% retention rate (*n* = 5,258). For the purposes of the current study, veterans who completed the Wave 6 survey comprised the study sample.  Table 1 contains Wave 6 sample characteristics.

More than one-third of the Wave 1 baseline TVMI sample (36.8%; *n* = 3516) indicated an interest in future research, and 3180 (33.2%) veterans completed the Wave 7 VETS survey administered in November 2020 (i.e., during the COVID-19 global pandemic), which was approximately 4-years postseparation. The Wave 8 VETS survey, completed by 2970 Veterans (31.0%), was administered in March 2023 (i.e., approximately two months after the Biden administration announced an end to the U.S. COVID-19 emergency in January 2023), which was approximately 6.5-years postseparation. Ethical approval for TVMI was obtained from ICF International, Inc., protocol number 151636.0.000.00.000. Ethical approval for VETS was obtained from (Anonymized University, protocol numbers 15732 and 21180). Informed consent was obtained prior to beginning the surveys.

### 2.2. Measures

#### 2.2.1. Psychological Symptoms

Psychological symptoms, the main outcome variable in this study, were measured using the 4-item version of the Patient Health Questionnaire (PHQ-4; [39]) at Waves 6, 7, and 8. The PHQ-4 asks respondents to indicate how often (not at all = 0, several days = 1, more than half the days = 2, and nearly every day = 3) over the prior 2 weeks they were bothered by symptoms of anxiety (e.g., persistent worry) and depression (e.g., feeling sad or down). PHQ-4 items were used as observed indicators of latent factors representing anxiety and depression at each wave. Model-based reliability ranged from 0.89 to 0.91 for the anxiety latent factor and 0.88–0.90 for the depression latent factor across the three waves. The anxiety and depression latent factors were significantly correlated within and between all timepoints (range = 0.55–0.86).

#### 2.2.2. Combat Exposure

Combat exposure was measured at Wave 1 (within the first 3 months of transitioning out of the military) with a 9-item scale developed from the Deployment Risk and Resilience Inventory-2 [40]. Respondents report how often they experienced combat-related events (e.g., engaging in battle, observing the death of a comrade, and firing a weapon). The measure also asks about exposure to the aftermath of combat (e.g., seeing wounded civilians and destroyed neighborhoods). Response options included 0 (never), 1 (once or twice), 2 (several times), and 3 (many times). Items were summed to create a total combat exposure score (Table 1; Cronbach's alpha = 0.94)

#### 2.2.3. Deployment Characteristics

Veterans were asked at Wave 1 to report their total number of combat deployments and the longest deployment, in months, they experienced while in the military.  Table 1 contains descriptive information for these deployment variables. For maximum deployment length, values ≥ 30 months (*n* = 7) were determined to be univariate outliers, and these values were winsorized to the highest nonoutlying value of 24 months.

#### 2.2.4. ACEs

ACEs were measured at Wave 5 with a 7-item measure developed by LeardMann and colleagues (2010) for a health-related study of marine recruits. Participants reported if they had experienced physical abuse, domestic violence, sexual abuse, neglect, and other difficult circumstances before the age of 17 using a binary yes (1) or no (0) scale. Previous research has produced evidence that supports the validity of the 7-item measure in a veteran sample [16]. Items were summed to create a total ACEs score (Table 1; Cronbach's alpha = 0.83).

#### 2.2.5. Gender and Race/Ethnicity

At Wave 1, veterans were asked to indicate their gender and race/ethnicity (Table 1, for sample details at Wave 6 [2.5-years post-separation]).

### 2.3. Data Analytic Approach

Confirmatory factor analysis (CFA) was used. CFA is suited to examining between-group differences and within-group changes over time in multiple constructs because it accounts for measurement error and allows for measurement invariance (i.e., construct equivalence across groups and/or time) to be directly tested rather than simply assumed [41]. Furthermore, CFA is easily extended to include observed or latent covariates [41]. A further advantage of CFA is the ability to use fit indices [42]: comparative fit index (CFI; ≥0.95), root mean square error of approximation (RMSEA; ≤0.06), and standardized root mean squared residual (SRMR; ≤0.08).

Three general models were tested. First, a longitudinal 6-factor (i.e., 2 factors × 3 waves) correlated CFA model was specified for the entire sample. Measurement invariance constraints (i.e., configural model: no constraints; metric model: constrained factor loadings; scalar model: constrained item intercepts) were sequentially imposed, and models were compared to investigate construct equivalence, which is a necessary condition for latent mean comparisons [43]. Measurement invariance was established by examining change in CFI (≥−0.01; [44]) and the Bayesian information criterion (BIC; <|2|; values exceeding |2| provide evidence favoring the model with the lower BIC; [45]). Wave 6 was set as the reference period (i.e., latent means constrained to zero) to allow for the calculation of latent mean differences over time. To examine changes from during to after COVID-19, Wave 7 was set as the reference period. Second, the longitudinal 6-factor correlated CFA model was extended to include multiple-group comparisons (i.e., White males and females and males and females from racial and ethnic minority groups) [41]. Measurement invariance constraints were sequentially imposed, and models were compared to examine construct equivalence using the same thresholds previously described. White males were set as the reference group at each wave to examine latent mean differences between groups. For within-group comparisons, Wave 6 was set as the reference period to examine before to during and before to after COVID-19 changes. Wave 7 was set as the reference period to examine during to after COVID-19 changes. Finally, the multiple-group longitudinal 6-factor correlated CFA model was extended by including ACEs, combat exposure, number of deployments, and maximum deployment length as covariates. Moderation was examined by comparing the magnitude of the covariates' effects across groups at each wave.

All CFA models were estimated using the lavaan package (v0.6-17; [46]) within the R environment (v4.3.3; [47]) using full information maximum likelihood (FIML) to accommodate missing data. Item-level missingness for depression and anxiety indicators was minimal at Wave 6 (0%–0.10%), Wave 7 (0.30%–0.60%), and Wave 8 (0%–0.10%). Missing data were also minimal (0.10%) for all covariates except ACEs (15.7%). The higher proportion of missingness for ACEs is due to the items being asked at the Wave 5 survey rather than the Wave 1 baseline survey.

FIML assumes data are missing at random. To increase the likelihood that data are missing at random, Wave 1 baseline variables that were found to correlate with missing data at Wave 8 were used as auxiliary variables in the saturated correlates approach [48]. Auxiliary variables included the following: age, paygrade, marital status, insurance coverage, national guard or reserve status, part-time college student status, medical-discharge status, and presence of a geocodeable mailing address. The measEq.syntax function of the semTools package (v0.5-6.928; [49]) was used to generate syntax that correctly constrained the longitudinal and multiple-group longitudinal CFAs for proper model identification. Residual correlations among the same observed indicators at each wave were allowed. The Wave 6 survey weight, which helps to adjust the sample to more closely match the population from which it was drawn and accounts for study attrition, was used in all the CFA models.

## 3. Results

### 3.1. Psychological Symptoms

Participants reported low levels of psychological symptoms over time. Mean depression scores for the full sample ranged from 1.24 at Wave 6–1.41 at Wave 8. Mean depression scores reported by male and female veterans from racial racial/ethnic minority groups were higher than the means reported by their White peers. The highest mean depression scores were reported by females from racial ethnic minorities, ranging from 1.82 to 1.84. Mean anxiety scores for the full sample ranged from 1.49 at Wave 6 to 1.70 at Wave 8. The highest anxiety scores were reported by female veterans (Ms = 1.59–2.10) and female veterans from racial/ethnic minorities (Ms = 2.19–2.51).

### 3.2. Data-Model Fit Analyses: Longitudinal and Multiple-Group Measurement Invariance

The top half of Table 2 shows the results of the longitudinal measurement invariance tests of the 6-factor correlated CFA model (i.e., depression and anxiety latent factors estimated at each wave). The CFI, RMSEA, and SRMR for the models reflected satisfactory fit to the data. Based on the change in CFI and BIC, the scalar invariant model (i.e., the model that constrains factor loadings and item requires equivalency across groups and time) was an adequate fit to the data (i.e., it fit at least as well as the metric invariant and configural models). Thus, the latent means of depression and anxiety can be meaningfully compared across the three waves of data [43].

The bottom half of Table 2 displays the results of the multiple groups (i.e., White males [*n* = 3026], males from racial/ethnic minorities [*n* = 1243], White females [*n* = 549], and females from racial/ethnic minorities [*n* = 421]) and longitudinal measurement invariance tests of the 6-factor correlated CFA model. Nineteen cases were excluded due to missing data on participants' race/ethnicity. The CFI, RMSEA, and SRMR values of the models indicated satisfactory fit. Change in CFI and BIC among the examined models indicated that the scalar invariant model represented a plausible fit to the data, and this facilitated a valid comparison of between- and within-group means before, during, and after COVID-19. The fit of the multiple-group longitudinal 6-factor correlated CFA model with covariates also had satisfactory fit (ꭙ^2^ [248, *n* = 5239] = 374.29, *p* < 0.001; CFI = 0.996; RMSEA [90% CI] = 0.029 [0.021, 0.036]; SRMR = 0.013).

### 3.3. Latent Mean Changes in Anxiety and Depression From Before to After COVID-19 for the Entire Sample

When looking at the total sample across time, depression and anxiety latent means were higher at Wave 7 (during COVID-19; depression: Δ*M* = 0.08, standard error (SE) = 0.02, *z* = 4.26, *p* < 0.001; anxiety: Δ*M* = 0.05, SE = 0.02, *z* = 2.81, *p*=0.005) and Wave 8 (after COVID-19; depression: Δ*M* = 0.14, SE = 0.02, *z* = 6.24, *p* < 0.001; anxiety: Δ*M* = 0.15, SE = 0.02, *z* = 6.54, *p* < 0.001) compared to Wave 6 (before COVID-19). Latent means for depression and anxiety were also found to be higher after COVID-19 (depression: Δ*M* = 0.06, SE = 0.02, *z* = 2.42, *p*=0.02; anxiety: Δ*M* = 0.10, SE = 0.02, *z* = 4.04, *p* < 0.001) compared to during COVID-19.

### 3.4. Mean Differences in Anxiety and Depression Pre-, Peri-, and Post-COVID-19: Between- Group Analysis

The top half of Table 3 displays results for between-group comparisons at each timepoint using White males as the reference group. The latent means for depression were higher for male and female veterans from racial/ethnic minorities compared to White male veterans before, during, and after COVID-19. No differences were observed between White male and female veterans' depression symptom latent means. For anxiety, latent means were higher for male and female veterans from racial/ethnic minorities and White female veterans compared to White male veterans before, during, and after COVID-19.

### 3.5. Changes in Anxiety and Depression From Pre- to Post-COVID-19: Within-Group Analyses

The results in the bottom half of Table 3 indicate within-group comparisons using before COVID-19 as the reference period (i.e., before to during COVID-19 and before to after COVID-19 differences). Within-group depression latent means increased from before to during COVID-19 and before to after COVID-19 for all gender and race/ethnicity groups. There was one exception to this finding. Depression symptom latent means for female veterans from racial/ethnic minorities did not change over time. Differences from during to after COVID-19 in the latent means of depression were only found for male veterans from racial/ethnic minorities (Δ*M* = 0.10, SE = 0.05, *z* = 2.06, *p*=0.04).

With respect to anxiety (Table 3), White females were the only group in which latent mean differences were found before and during COVID-19 and before and after COVID-19; however, no during to after COVID-19 differences were found for White females. No latent mean differences were observed for female veterans from racial/ethnic minorities. Latent mean differences were found for White males and males from racial/ethnic minorities from before to after COVID-19 and during to after COVID-19 (White males: Δ*M* = 0.12, *SE* = 0.03, *z* = 3.92, *p* < 0.001; male Veterans from racial/ethnic minorities: Δ*M* = 0.12, SE = 0.05, *z* = 2.26, *p*=0.02).

### 3.6. Effect of Combat, ACEs, and Deployment Characteristics on Anxiety and Depression Over Time

Table 4 illustrates the effects of the covariates on depression and anxiety. Combat exposure and ACEs were positively associated with depression and anxiety symptoms at all time points for male veterans. The number of deployments was associated with decreased anxiety and depression symptoms among White male veterans at all time points. Thus, White male veterans with more deployments had lower symptoms of depression and anxiety at all time points compared to White male veterans with fewer deployments after controlling for combat exposure, ACEs, and maximum deployment length. This effect was also found in anxiety among male veterans from racial/ethnic minorities after COVID-19. Maximum deployment length was positively associated with anxiety and depression symptomology before and during COVID-19 for male veterans from racial/ethnic minorities. For White females, combat exposure was positively associated with depression symptoms before COVID-19 and with anxiety symptoms at each time point, while ACEs were positively associated with anxiety symptoms at all time points. Number of deployments was not associated with symptoms of depression but was negatively associated with anxiety symptoms before COVID-19 among White female veterans. There was no association between maximum length of deployment for White female veterans. Among female veterans from racial/ethnic minorities, combat exposure was associated with increased anxiety symptoms before COVID-19 but had no association with symptoms of depression. ACEs were positively associated with depression symptoms before and during COVID-19 and with anxiety symptoms before COVID-19 among female veterans from racial/ethnic minorities. Neither the number nor length of deployment was associated with psychological symptoms among female veterans from racial/ethnic minorities.

## 4. Moderation Effects

Between-group differences for the covariates' effects before, during, and after COVID-19 were investigated to explore the possibility of moderation (e.g., the effect of ACEs on before COVID-19 depression depends on one's gender and race/ethnicity combination). After adjusting *p*-values via the false discovery rate (FDR) method due to multiple testing (i.e., six group comparisons × four covariates for depression and anxiety at each time point), the only statistically significant moderation effect found was for the influence of maximum deployment length on anxiety during COVID-19. The effect for male veterans from racial/ethnic minorities was found to be greater than the effect for White males (Δ*B* = 0.04, SE = 0.01, *z* = 3.14, *p*=0.04, Δ*β* = 0.20).

## 5. Discussion

This was the first study to examine how symptoms of anxiety and depression changed over time before, during, and after COVID-19 among a large and diverse sample of male and female post-9/11 veterans. Mean depression and anxiety scores fell within the normal range over time. The lack of elevated symptom reporting may be because this was not a clinical sample. Another possible explanation for the low mean scores on depression and anxiety is self-report bias. Military culture conditions veterans to deny personal problems, appear stoic and in control, and present themselves as invincible [50]. As a result, they may find it difficult to admit to or understate their mental health problems. On the other hand, it must also be noted that those in the military are trained to be psychologically resilient in times of crisis and chaos [51] and this training may have been helpful to veterans during the pandemic. Nonetheless, at all points in time, depression, and anxiety scores were highest among female veterans from racial/ethnic minorities, which replicates some prior research [52]. Male veterans from racial/ethnic minorities had higher mean depression scores than their White male peers, which also replicates prior findings [53].

When looking at the entire sample of veterans, depression, and anxiety symptoms were significantly higher during and after COVID-19 compared to before COVID-19. Depression and anxiety symptoms after COVID-19 were significantly higher than during COVID-19. These results should not be surprising. The COVID-19 pandemic led to substantial alterations in day-to-day life, economic turndowns, high levels of illness and death, and fear about the future [54] These COVID-19-related impacts were not short-lived and continued after the pandemic was declared over [55, 56] and, hence, may account for the higher symptom levels from during to after COVID-19 for some veterans.

A few other studies have examined changes in mental health symptoms during COVID-19 among veterans. One study found that positive screens for generalized anxiety disorder increased from before to during COVID-19, but screens for PTSD and depression did not change [57]. Similarly, positive depression screens did not differ from before to after COVID-19 among a sample of transitioning veterans who were participating in a job-search program [58]. On the other hand, a nationally representative study of veterans found that within the first year of COVID-19, the prevalence of positive screens for distress increased by 51%; however, within 2 years, the distress indicator had returned to pre-pandemic levels [59]. In this study, younger and female Veterans evidenced the steepest increases in distress. Another nationally representative study of veterans found little change in mental health symptoms pre- to during COVID-19 [60]. Slight increases in symptoms of PTSD, depression, and anxiety were reported among transitioning young adult veterans, and veterans from racial and ethnic minorities experienced higher symptom levels [61]. Among a large sample of veterans residing in community living centers, depression symptom scores did not change from before to several periods during COVID-19 [62]. The results of these studies vary which is likely due to differences in sampling and measurement. In addition, these studies were conducted with veterans of all wars.

The hypothesis of the current study that female veterans and veterans from racial and ethnic minority groups would report higher levels of anxiety and depression symptoms was partially supported. Compared to White male veterans, male and female veterans from racial and ethnic minority groups reported higher depression and anxiety symptoms over time. White male and female veterans did not differ in depression, but White females reported higher symptoms of anxiety. Previous research has shown that veterans from racial and ethnic minority groups reported higher levels of stress across numerous life domains relative to their White peers [1]. The accumulation of stress is positively correlated with mental health problems [63, 64]. In addition, racial and ethnic minority individuals experience a variety of daily stressors related to race/ethnicity that put them at risk for mental health challenges [65, 66], including physiological changes related to stress exposure, such as decreased telomere length [67]. Why White male and female veterans did not evidence differences in depression symptom levels at each timepoint is unclear. There is evidence that the prevalence of depressive symptoms of male and female veterans varies across studies depending on when the data was collected and what measures were used [68]. Perhaps female veterans, who are immersed within the hyper-masculine and self-sufficient military culture [69], may underreport some mental health problems (such as depression) due to help-seeking and mental health stigma [70].

In partial support of the study's prediction, depression symptoms increased from before to during and from before to after COVID-19 for White males and females and men from racial/ethnic minorities. Contrary to prediction, depression symptom scores did not change over time for women veterans from racial/ethnic minorities. At all points in time, however, female veterans from racial/ethnic minorities evidenced higher levels of depressive symptomology compared to the other groups. Hence, a parsimonious explanation for this finding is that there was a ceiling effect that left little room to move over time [71]. Depression symptoms only increased from during to after COVID-19 among male veterans from racial/ethnic minorities. The COVID-19 pandemic overlapped closely in time with several highly publicized cases of police shootings of Black males. These situations, and the ongoing distrust of the healthcare system, were elements that likely had a disproportionately strong impact on the stress and mental health of men from racial/ethnic minorities [72]. Other factors that have been theorized to contribute to mental health challenges for men from racial/ethnic minorities during and after COVID-19 was the disproportionate burden of the economic impact of COVID-19 on them, and the general slowness of the economic recovery [73].

Contrary to prediction, White females were the only group in which differences in anxiety symptoms were found both before to during COVID-19 and before to after COVID-19, however, not during to after COVID-19. Anxiety did not change over time for female veterans from racial/ethnic minorities. Increases in anxiety symptoms were found for White male veterans and male veterans from racial/ethnic minorities from before to after COVID-19 and during to after COVID-19. Again, the findings that female veterans from racial/ethnic minorities did not evidence changes in anxiety symptoms over time was striking. This finding could also reflect a ceiling effect. On the other hand, an argument can be made that women from racial/ethnic minorities, particularly Black women, can be viewed from a strengths-based perspective. Black women report relatively lower rates of mental disorders compared to White women and, in some cases, Black men [74]. In addition, research has shown that Black women possess psychological resources that are health protective [75] and include social support, self-esteem, and mastery [76].

In line with accumulation theory [63], combat exposure and ACEs were generally found to have positive and statistically significant associations with depression and anxiety symptoms across time. However, for female veterans from racial/ethnic minorities, combat exposure and ACEs had more circumspect associations with symptoms. Perhaps there was insufficient power to detect these associations for female veterans from racial/ethnic minorities as the sample size was limited. Contrary to accumulation theory, but in line with a recent study [77], the number of deployments for White male veterans was associated with lower depression and anxiety symptom scores across all time periods. The number of deployments was not related to depression symptoms over time for veterans from racial/ethnic minorities. Among White females and male veterans from racial/ethnic minorities, the number of deployments was inconsistently related to lower anxiety symptomology. These findings suggest that the number of deployments may have built a degree of resilience that perhaps prepared veterans to handle the challenges associated with COVID-19, especially White veterans. Future studies should examine the number of deployments as a potential protective factor for mental health problems, particularly among veterans from racial/ethnic minorities given the inconsistent findings in this study. Stress inoculation theory posits that exposure to stress can help individuals develop psychological resilience [78]. In the face of stress, individuals may undergo psychological changes to accommodate and manage stressors more adeptly [78]. Military service, while challenging and stressful, can build Service members' competence, coping skills, and courage. Indeed, individuals within the military often demonstrate resilience even in the face of life-threatening and traumatic situations [79]. Maximum deployment length was unrelated to depression and anxiety symptoms among White males and females and female veterans from racial/ethnic minorities. For male veterans from racial/ethnic minorities, maximum deployment length was associated with higher levels of depression and anxiety symptoms before and during COVID-19. Few studies have examined the impact of deployment length. Some evidence suggests that a mismatch between expected and actual length of deployment is associated with poorer mental health [80].

### 5.1. Study Limitations

This study had limitations. First, while the Wave 1 sample was representative of the population of service members leaving active duty in 2016, the generalizability of the current study is not clear due to attrition of participants over time. Second, respondents relied on retrospection to report ACEs and combat experiences. Third, the timing of the before and after COVID-19 data collection was reasonable based on when the pandemic began and ended. However, the timing of the after COVID-19 data collection occurred on the cusp of when the COVID-19 emergency in the United States was declared over. Fourth, any number of unmeasured variables could have influenced depression and anxiety symptoms over time. These could include family composition, employment status, quality of housing and neighborhood safety, and access to healthcare. The results of the current study, comprised only of post-9/11 veterans, should not be generalized to other veterans. Finally, the study did not examine respondents' use of coping strategies, and it did not assess how effectively copies strategies were used. Despite these limitations, the current study found that depressive and anxious symptom levels increased in post-9/11 veterans from before to after COVID-19. In addition, the study outcomes indicate that gender, race/ethnicity, ACES, combat exposure, and number and length of deployments impacted veterans' mental health experiences during that timeframe.

## 6. Conclusion

Future global pandemics are predicted to occur, and these are likely to be as detrimental as the COVID-19 pandemic, perhaps even worse [81]. One of the important lessons learned in the aftermath of the COVID-19 pandemic was that race and ethnicity played important roles in predicting health outcomes in the general public. This study confirms that finding to be true among a large sample of United States post-9/11 veterans.

## Figures and Tables

**Table 1:** Wave 6 sample characteristics (before COVID-19; *n* = 5258).

Demographic	*n*	%
Gender		
Male	4287	81.5
Female	971	18.5
Race/ethnicity^a^		
White, non-Hispanic	3575	68.2
Black, non-Hispanic	497	9.5
Hispanic	692	13.2
AHPI, non-Hispanic	206	3.9
Other race, non-Hispanic	269	5.1
Service branch/component^b^		
Army	1674	31.9
Navy	1013	19.3
Air force	1044	19.9
Marine corps	871	16.6
National guard/reserves	653	12.4
Paygrade		
E1–E4	1552	29.5
E5–E6	1569	29.8
E7–E9	877	16.7
W1–W5	77	1.5
O1–O3	473	9.0
O4–O10	710	13.5

	**Mean (SD)**	**Range**

Age (years)^c^	35.95 (9.43)	20.50–66.50
ACEs^d^	1.20 (1.83)	0–7
Combat exposure^e^	4.96 (7.16)	0–27
Number of deployments^f^	1.81 (2.05)	0–10
Maximum deployment length^e^	6.35 (5.10)	0–24
Depression symptoms^g^	1.24 (1.65)	0–6
Anxiety symptoms^h^	1.49 (1.80)	0–6

*Note:* percentages may not add to 100% due to rounding error. E1–E4, junior enlisted; E5–E6, mid-grade enlisted; E7–E9, senior enlisted; O1–O3, junior officers; O4–O10, senior officers.

Abbreviations: ACEs, adverse childhood experiences; AHPI, Asian, Hawaiian, Pacific Islander; SD, standard deviation.

^a^ *n* = 5239.

^b^ *n* = 5255.

^c^ *n* = 5168.

^d^ *n* = 4434.

^e^ *n* = 5253.

^f^ *n* = 5254.

^g^ *n* = 5252.

^h^ *n* = 5256.

**Table 2:** Measurement invariance model comparisons.

6-Factor correlated longitudinal invariance models							
Model	*χ* ^2^ (df, *n*)	CFI	RMSEA (90% CI)	SRMR	BIC	ΔCFI	ΔBIC
Configural	50.83 (27, 5258), *p*=0.004	0.999	0.023 (0.011, 0.033)	0.006	170,590.03	—	—
Metric	56.28 (31, 5258), *p*=0.004	0.999	0.021 (0.011, 0.031)	0.006	170,562.37	0	−27.66
Scalar	68.79 (35, 5258), *p*=0.001	0.998	0.023 (0.014, 0.032)	0.006	170,545.30	−0.001	−17.07

**6-Factor correlated multiple group and longitudinal invariance models**							
**Model**	***χ* ^2^ (df, *n*)**	**CFI**	**RMSEA (90% CI)**	**SRMR**	**BIC**	**ΔCFI**	**ΔBIC**

Configural	136.92 (108, 5239), *p*=0.03	0.999	0.022 (0.000, 0.036)	0.009	166,075.60	—	—
Metric	174.80 (130, 5239), *p*=0.005	0.998	0.025 (0.010, 0.036)	0.013	165,933.21	−0.001	−142.39
Scalar	238.26 (152, 5239), *p* < 0.001	0.997	0.032 (0.022, 0.041)	0.014	165,821.90	−0.001	−111.31

*Note:* Yuan–Bentler correction applied to chi-squared statistic. For CFI and RMSEA, robust values are reported. Wave 6 sampling weights applied, and auxiliary variables included. 19 participants excluded from the multiple-group analyses due to missing data on race/ethnicity. —, not applicable.

Abbreviations: BIC, Bayesian information criterion; CFI, comparative fit index; RMSEA, root mean squared error of approximation; SRMR, standardized root mean squared residual.

**Table 3:** Mean differences between and within groups before, during, and after COVID-19.

Comparisons	Before COVID-19 (Wave 6) depression			During COVID-19 (Wave 7) depression			After COVID-19 (Wave 8) depression			Before COVID-19 (Wave 6) anxiety			During COVID-19 (Wave 7) anxiety			After COVID-19 (Wave 8) anxiety		
Between-group	Δ*M*	SE	*p*	Δ*M*	SE	*p*	Δ*M*	SE	*p*	Δ*M*	SE	*p*	Δ*M*	SE	*p*	Δ*M*	SE	*p*
																		
White males (reference group)	0	—	—	0	—	—	0	—	—	0	—	—	0	—	—	0	—	—
Males from racial/ethnic minorities	**0.22**	**0.05**	**<0.001**	**0.22**	**0.06**	**<0.001**	**0.27**	**0.06**	**<0.001**	**0.16**	**0.04**	**<0.001**	**0.24**	**0.06**	**<0.001**	**0.22**	**0.06**	**<0.001**
White females	0.04	0.06	0.50	0.13	0.07	0.08	0.06	0.07	0.40	**0.15**	**0.06**	**0.01**	**0.39**	**0.08**	**<0.001**	**0.30**	**0.08**	**<0.001**
Females from racial/ethnic minorities	**0.52**	**0.08**	**<0.001**	**0.42**	**0.09**	**<0.001**	**0.34**	**0.10**	**0.001**	**0.54**	**0.07**	**<0.001**	**0.66**	**0.09**	**<0.001**	**0.47**	**0.09**	**<0.001**

**Comparisons**	**Before COVID-19 (Wave 6) depression (reference period)**			**During COVID-19 (Wave 7) depression**			**After COVID-19 (Wave 8) depression**			**Before COVID-19 (Wave 6) anxiety (reference period)**			**During COVID-19 (Wave 7) anxiety**			**After COVID-19 (Wave 8) anxiety**		
**Within-group**	**Δ*M***	**SE**	***p***	**Δ*M***	**SE**	***p***	**Δ*M***	**SE**	***p***	**Δ*M***	**SE**	***p***	**Δ*M***	**SE**	***p***	**Δ*M***	**SE**	***p***

White males	0	—	—	**0.09**	**0.03**	**0.002**	**0.14**	**0.03**	**<0.001**	0	—	—	0.01	0.03	0.82	**0.13**⁣^*∗∗∗*^	**0.03**	**<0.001**
Males from racial and ethnic groups	0	—	—	**0.09**	**0.04**	**0.03**	**0.19** *⁣* ^*∗*^	**0.05**	**<0.001**	0	—	—	0.07	0.04	0.06	**0.19** *⁣* ^*∗*^	**0.05**	**<0.001**
White females	0	—	—	**0.16**	**0.06**	**0.01**	**0.15**	**0.06**	**0.01**	0	—	—	**0.22**	**0.06**	**<0.001**	**0.27**	**0.07**	**<0.001**
Females from racial and ethnic groups	0	—	—	0.01	0.06	0.84	−0.01	0.08	0.93	0	—	—	0.09	0.07	0.16	0.07	0.08	0.40

*Note:* For between-group latent mean comparisons at each wave, white males' latent means for depression and anxiety are set to zero for model identification (latent mean differences are read from top to bottom). For within-group latent mean comparisons across waves, the Wave 6 latent means for depression and anxiety are set to zero for model identification (latent mean differences are read from left to right). Δ*M* = Latent mean difference (positive values represent an increase and negative values represent a decrease in latent means when constraining the latent mean of the reference group or period to 0); — = not estimated by the model. Numbers in bold are statistically significant.

Abbreviations: SE = standard error; *p*= *p*-value.

*⁣* ^*∗∗∗*^Latent mean difference during to after COVID-19 is statistically significant at *p* < 0.001 (Wave 7 set as the reference period; specific values not shown due to table space constraints).

*⁣* ^*∗*^Latent mean difference during to after COVID-19 is statistically significant at *p* < 0.05 (Wave 7 set as the reference period; specific values not shown due to table space constraints).

**Table 4:** Effects of combat exposure, ACEs, number of deployments, and maximum deployment length on depression and anxiety before, during, and after COVID-19.

Group and variables	Before COVID-19 (Wave 6) depression				During COVID-19 (Wave 7) depression				After COVID-19 (Wave 8) depression			
	*B*	SE	*p*	*β*	*B*	SE	*p*	*β*	*B*	SE	*p*	*β*
White males												
Combat exposure	**0.02**	**0.004**	**<0.001**	**0.16**	**0.02**	**0.01**	**<0.001**	**0.14**	**0.02**	**0.01**	**0.002**	**0.11**
ACEs	**0.16**	**0.02**	**<0.001**	**0.26**	**0.18**	**0.02**	**<0.001**	**0.29**	**0.17**	**0.02**	**<0.001**	**0.26**
Deployment number	**−** **0**.**04**	**0.01**	**<0.001**	**−** **0**.**08**	**−** **0**.**06**	**0.01**	**<0.001**	**−** **0**.**11**	**−** **0**.**05**	**0.02**	**0.001**	**−** **0**.**09**
Maximum deployment length	−0.01	0.01	0.12	−0.04	−0.01	0.01	0.38	−0.03	−0.003	0.01	0.73	−0.01
Males from racial/ethnic minorities												
Combat exposure	**0.02**	**0.01**	**0.002**	**0.13**	**0.02**	**0.01**	**0.048**	**0.11**	**0.02**	**0.01**	**0.01**	**0.13**
ACEs	**0.15**	**0.03**	**<0.001**	**0.23**	**0.13**	**0.04**	**<0.001**	**0.21**	**0.14**	**0.04**	**<0.001**	**0.19**
Deployment number	−0.04	0.02	0.07	−0.06	−0.04	0.03	0.21	−0.06	−0.06	0.04	0.09	−0.08
Maximum deployment length	**0.02**	**0.01**	**0.01**	**0.11**	**0.02**	**0.01**	**0.04**	**0.12**	0.003	0.01	0.83	0.01
White females												
Combat exposure	**0.04**	**0.02**	**0.03**	**0.13**	−.003	0.02	0.90	−.01	0.02	0.02	0.27	0.07
ACEs	**0.17**	**0.03**	**<0.001**	**0.30**	**0.18**	**0.04**	**<0.001**	**0.32**	**0.16**	**0.03**	**<0.001**	**0.30**
Deployment number	−0.01	0.05	0.83	−0.01	−0.03	0.05	0.53	−0.04	−0.09	0.05	0.06	−0.11
Maximum deployment length	0	0.02	1	0	0.01	0.02	0.78	0.02	0.01	0.02	0.67	0.03
Females from racial/ethnic minorities												
Combat exposure	0.03	0.02	0.17	0.08	−0.01	0.03	0.66	−0.03	0.02	0.03	0.50	0.05
ACEs	**0.12**	**0.04**	**0.002**	**0.20**	**0.10**	**0.04**	**0.01**	**0.19**	0.06	0.05	0.20	0.11
Deployment number	−0.08	0.05	0.14	−0.07	−0.07	0.06	0.24	−0.07	0.003	0.07	0.97	0.003
Maximum deployment length	0.01	0.02	0.79	0.02	0.04	0.02	0.11	0.15	0.03	0.02	0.20	0.11

**Group and variables**	**Before COVID-19 (Wave 6)** **anxiety**				**During COVID-19 (Wave 7)** **anxiety**				**After COVID-19 (Wave 8)** **anxiety**			
	***B***	**SE**	***p***	***β***	***B***	**SE**	***p***	***β***	***B***	**SE**	***p***	***β***

White males												
Combat exposure	**0.03**	**0.004**	**<0.001**	**0.19**	**0.02**	**0.004**	**<0.001**	**0.15**	**0.02**	**0.01**	**<0.001**	**0.15**
ACEs	**0.16**	**0.02**	**<0.001**	**0.26**	**0.14**	**0.02**	**<0.001**	**0.23**	**0.16**	**0.02**	**<0.001**	**0.24**
Deployment number	**−** **0**.**04**	**0.01**	**<0.001**	**−** **0**.**08**	**−** **0**.**05**	**0.01**	**<0.001**	**−** **0**.**10**	**−** **0**.**05**	**0.02**	**0.003**	**−** **0**.**09**
Maximum deployment length	−0.01	0.01	0.09	−0.04	−0.004	0.01	0.49	−0.02	−0.01	0.01	0.35	−0.03
Males from racial/ethnic minorities												
Combat exposure	**0.02**	**0.01**	**<0.001**	**0.16**	**0.02**	**0.01**	**0.03**	**0.11**	**0.03**	**0.01**	**0.003**	**0.18**
ACEs	**0.13**	**0.02**	**<0.001**	**0.21**	**0.09**	**0.02**	**0.004**	**0.14**	**0.11**	**0.03**	**0.002**	**0.16**
Deployment number	−0.02	0.02	0.34	−0.03	−0.05	0.03	0.05	−0.09	−0.**07**	**0.03**	**0.02**	−0.**11**
Maximum deployment length	**0.02**	**0.01**	**0.02**	**0.11**	**0.04**	**0.01**	**0.001**	**0.18**	0.02	0.01	0.13	0.09
White females												
Combat exposure	**0.05**	**0.02**	**0.004**	**0.15**	**0.02**	**0.02**	**0.44**	**0.05**	0.003	0.02	0.88	0.01
ACEs	**0.18**	**0.03**	**<0.001**	**0.33**	**0.13**	**0.04**	**0.001**	**0.23**	**0.16**	**0.04**	**<0.001**	**0.29**
Deployment number	−**0**.**06**	**0.03**	**0.03**	−**0**.**07**	−0.08	0.04	0.06	−0.09	−0.09	0.05	0.09	−0.10
Maximum deployment length	−0.01	0.01	0.45	−0.04	0.01	0.02	0.65	0.03	0.01	0.02	0.48	0.06
Females from racial/ethnic minorities												
Combat exposure	**0.04**	**0.02**	**0.046**	**0.13**	0.02	0.03	0.53	0.05	0.02	0.03	0.58	0.05
ACEs	**0.13**	**0.03**	**<0.001**	**0.24**	0.03	0.04	0.38	0.07	0.02	0.05	0.61	0.04
Deployment number	−0.08	0.05	0.13	−0.09	−0.12	0.07	0.06	−0.13	0.003	0.07	0.97	0.003
Maximum deployment length	−0.001	0.02	0.96	−0.004	0.02	0.02	0.31	0.10	0.001	0.02	0.95	0.01

*Note:* White males, *n* = 3026; males from racial/ethnic minorities, *n* = 1243; white females, *n* = 549; females from racial/ethnic minorities *n* = 421. *B*, unstandardized regression coefficient. *β*, standardized regression coefficient. Statistically significant effects (*p* < 0.05) are bolded.

Abbreviations: ACEs, adverse childhood experiences; SE, standard error.

## Data Availability

The TVMI data gathered from Waves 1–6 (ICPSR 38051) are available at ICPSR https://www.icpsr.umich.edu/web/ICPSR/studies/38051/publications. Veterans Engaging in Transitions Studies (VETS) Waves 7–8 data are not yet publicly available but will be available at ICPSR within the next 18 months.
